# Assessing the Feasibility of Retrospective and Prospective Clinical Audit in Farm Animal Veterinary Practice

**DOI:** 10.3390/vetsci8040062

**Published:** 2021-04-13

**Authors:** Katie Waine, Constance White, Rachel S. Dean, Chris Hudson, Jonathan N. Huxley, Marnie L. Brennan

**Affiliations:** 1 School of Veterinary Medicine and Science, Sutton Bonington Campus, University of Nottingham, Loughborough LE12 5RD, UK; katie.waine@nottingham.ac.uk (K.W.); chris.hudson@nottingham.ac.uk (C.H.); 2 Carlson College of Veterinary Medicine, Oregon State University, Corvallis, OR 97331, USA; whitecon@oregonstate.edu; 3 VetPartners Ltd., Leeman House, Station Business Park, Holgate Park Drive, York YO26 4GB, UK; Rachel.Dean@vetpartners.co.uk; 4 School of Veterinary Science, Massey University, Private Bag 11 222, Palmerston North 4442, New Zealand; j.huxley@massey.ac.nz; 5 Centre for Evidence-Based Veterinary Medicine, School of Veterinary Medicine and Science, Sutton Bonington Campus, University of Nottingham, Loughborough LE12 5RD, UK

**Keywords:** clinical audit, farm animal practice, farm animal veterinary medicine, quality improvement, retrospective clinical audit, prospective clinical audit

## Abstract

As a quality improvement tool, clinical audit has been extensively described in the medical literature. There is scant literature on the use of clinical audit in the farm animal veterinary setting. This study describes the process and feasibility of prospective and retrospective data collection for farm animal clinical audit performed at three different farm animal practices in the United Kingdom. Retrospective clinical audit was difficult in all three practices due to barriers in establishing diagnosis and patient identity from clinical records. Prospective data collection proved feasible but depended on adequate case accrual and practitioner engagement. The decision to conduct clinical audit retrospectively or prospectively will depend on the topic to audit, the availability of data and the wishes of the team members involved. Further work is required looking at the use of clinical coding and alternatives to using practice management software to improve retrospective data availability for clinical audit.

## 1. Introduction

Clinical audit is a quality improvement tool that can be used to measure process, structure, or outcomes of care provided by veterinary surgeons and practices [1,2]. Its use as a standard for clinical governance is encouraged in the United Kingdom (UK) by the Royal College of Veterinary Surgeons [3]. Clinical audit is considered to be a cyclical process in which patient care processes or outcomes are measured against explicitly defined quality criteria; results are then used to revise and implement changes in process or procedures of care with iterative re-evaluation of quality improvement on re-audit [4]. Clinical audit in human medicine typically relies on comparison of current practice or outcomes to well defined and evidence-based ‘gold standards’. In veterinary medicine, lack of evidence-based standards in many areas means that clinical audit may be done to compare practice with a consensus or opinion-based standard, or may be used to create standards or values that allow individual practices to benchmark against processes or outcomes of other practices [5,6].

Clinical audits can be conducted in a number of areas: process (e.g., adherence to protocols, delivery of services), outcomes (client or patient results), structure, or for a single critical event [7]. Collection of measurable data for any type of clinical audit is critical to audit success and can be done retrospectively, by examining data that already exists, or prospectively, with data collected at the time of clinical service delivery [8]. As retrospective clinical audit relies on pre-existing data, it may be performed more quickly but at the expense of data quality, and has been suggested to be more appropriate for historical benchmarking or in response to a critical incident [8]. However, the availability of suitable data may limit the ability to successfully execute the process [9]. Level of data existence may depend on the computer software utilised by the practice and the detail recorded by individual veterinary surgeons.

Although retrospective clinical audit may be most convenient, prospective clinical audit is required where sufficient data does not already exist on the desired topic. Prospective data collection allows for accurate, unambiguous data to be recorded by clinical staff at the point of care [8]. However, prospective data collection can increase clinical audit workload as well as obscure what is actual and routine clinical practice through the Hawthorne effect [10,11].

A number of small animal clinical audits have been described (reviewed by Rose and colleagues [6]). Retrospective clinical audits have most commonly been done on computerized individual patient records using key words, prescription data, or billing codes. Few farm animal clinical audits have been reported [9,12] and the process of conducting either retrospective or prospective clinical audit in farm animal veterinary practice is relatively unexplored. The aim of this study was to describe the implementation and feasibility of retrospective and prospective clinical audits in three veterinary practices in the United Kingdom providing farm animal care.

## 2. Materials and Methods

### 2.1. Participant Recruitment

Three veterinary practices from different geographic regions of the UK were contacted by the final author (MLB) to perform clinical audits in partnership with the Centre for Evidence-based Veterinary Medicine (CEVM). Each practice delivered farm animal care (predominantly or solely ruminants) either as a farm-animal exclusive or mixed practice. Study aims and information was provided via a mailed information sheet and followed up by telephone and email communication with the practice. All portions of this study and associated work received approval from the School of Veterinary Medicine and Science’s Clinical Ethical Review Panel at the University of Nottingham (approval number 906 130704).

### 2.2. Establishment, Monitoring, and Communication Systems of Clinical Audits

For each practice the first author (KW) acted as the coordinator, assisting with the logistics and data analysis but within each practice, a clinician acted as the clinical audit lead. An initial in-person meeting (‘priority setting meeting’, PSM) was held by the researchers at each individual veterinary practice using an amended James Lind Alliance Priority Setting Partnership Meeting format [13]. Meetings were held at the close of working hours to optimize opportunity for attendance. Prior to this meeting, team members had been asked to submit topic ideas which were collated into a list. Final topics were selected by each practice’s PSM attendees using an adapted nominal group technique [14] with final selection by consensus or vote during the PSM or later by electronic means; options for data collection and ongoing communication were also discussed during the meetings. A consent form assuring practice and practitioner anonymity, as well as the voluntary nature of study participation, was completed by each veterinarian at the initial meeting. Subsequent in-person meetings with the researchers occurred after the first round of data collection, and at the end of the project. Meetings were recorded using a Dictaphone with participant consent; these were transcribed and assessed by the researchers using a thematic approach using Microsoft Excel V.14.0.6 software (2010 Microsoft Corporation, Redmond, Washington, DC, USA). Participants also completed an individual questionnaire on demographics, education, prior experience with and attitudes toward clinical audit. Background information on each practice was obtained from a partner or director at the meeting via questionnaire regarding staffing, practice standards, case mix, billing, and practice management software (PMS) characteristics. Practice clients were sent a letter or newsletter describing the study with voluntary opt-out instructions.

Minutes from the meetings were emailed to the practices within a few days and included a summary of the meeting and discussion, any supporting documents and the plan moving forward. Email was also used to advise on changes or updates, collect votes for decision making, ask for opinions, promote discussion and as reminders for data collection. A quarterly newsletter was created and sent out to all three practices involved in the project. The newsletter provided an update on how the work was progressing as well as conference and publication news.

### 2.3. Clinical Audit Standards Identification

The literature was searched for any existing standards or evidence relevant to topics chosen by the practices. CAB Abstracts (1910–2014) and Medline (1946–2014) were searched using keywords and subject headings, as the combination of both provides the best coverage of veterinary related content [15]. Inclusion and exclusion criteria were applied to identify relevant papers; auto alerts were set up for all searches to ensure new relevant literature was identified as the study progressed.

### 2.4. Retrospective Data Collection

Each practice was visited by KW to collect information regarding practice systems for clinical information recording, storage, and utilization. Data collection methods were examined with assistance from a nominated member of the practice team; data flow was assessed from the time that farmers telephoned the practice to arrange a visit, through to billing.

When feasible, retrospective data was collected and collated for each practice’s clinical audit topic for January–December 2013. Collection and collation was conducted by KW, with assistance from the practice staff member in each practice.

### 2.5. Prospective Data Collection

Data were collected prospectively by the veterinary surgeons within their practices for a pre-set period of time. This was initially suggested as 12 months in each practice.

Participating practices selected their preferred data collection method from a number of proposed options; all chose paper forms carried in a bound paper data collection booklet. Data collection forms were carried by the individual veterinary surgeons in practices 1 and 2, whilst practice 3 opted to place the booklets in ambulatory surgical kits. Data collection forms were pre-tested by University of Nottingham farm animal and CEVM clinicians and then further amended after initial pilot studies using participant feedback.

Practice 1 and Practice 2 transferred the information from the booklets to an online form (SurveyMonkey Inc., San Mateo, CA, USA). In Practice 3, the completed data collection forms were removed from the surgical kits by a practice veterinary nurse and were then processed by one veterinary surgeon who entered the information into Microsoft Excel V.14.0.6 (2010 Microsoft Corporation, Redmond, WA, USA). The follow-up information in Practice 3 was collected from the milk recording data (Interherd, National Milk Records PLC, Chippenham, Wiltshire, UK) in batches on regular occasions throughout the audit by the allocated veterinary surgeon in the practice.

### 2.6. Data Analysis

Data collected from the retrospective and prospective clinical audit process were transferred to Microsoft Excel V.14.0.6 (2010 Microsoft Corporation, Redmond, Washington, DC, USA) by KW and analyzed descriptively by KW and CW. Descriptive statistics were performed with Stata IC 16 (StataCorp, College Station, TX, USA); data was tested for normality using the Shapiro–Wilk test; normal data is presented as mean with standard deviation whilst non-normal data is presented as median and interquartile range (IQR).

## 3. Results

### 3.1. Practice and Veterinarian Questionnaires

All three practices were members of the RCVS Practice Standards Scheme at the General Practice level, and all charged for their work by time spent on the farm (as opposed to fee-per-task). In total, twenty-three veterinarians between the three practices (median 8 surgeons per practice) conducted either mixed species or farm animal-exclusive work and included senior and junior staff. Nineteen veterinarians completed the individual questionnaire. Median time in practice was 9 years (IQR 4-18); fourteen held or were currently undertaking advanced practice qualifications (RCVS certificate, RCVS or European Diploma). Sixteen veterinarians indicated they had previously participated in a clinical audit and all respondents agreed or strongly agreed that clinical audit could improve practice standards. Most (12/19) believed that clinical audit should be compulsory for farm animal practice but a substantial number (8/19) agreed with a statement ‘clinical audit takes up a large amount of time’. Fourteen respondents agreed that they understood the concept of clinical audit, however free text descriptions of clinical audit varied between respondents: 10/19 described assessment or measurement of veterinary care, 15/19 mentioned monitoring of outcomes, 9/19 described measurement of clinical performance, 6/19 proposed measurement against protocols, benchmarks, or published data. None of the respondents referenced a cyclical process. Perceived barriers to clinical audit mentioned by two or more respondents were time (8/19), data accuracy (6/19), participant engagement (4/19), follow-up data collection (4/19), and diagnostic specification (2/19). Comments recorded during the PSM paralleled these themes with concerns about data quality and source, case definitions, and sufficient caseload for audit topic.

### 3.2. Clinical Audit Topic Selection

Not all veterinary surgeons performing farm animal work were able to attend the initial meeting: 4/8 vets attended the PSM at Practice 1; 4/6 at Practice 2 and 6/9 at Practice 3.

Multiple topics were nominated by veterinarians in each practice with a number of suggested topics in common between practices (Table 1). Development of clear case criteria, predicted case accrual, and feasibility of data collection for each topic were considered. Clinical audits of clinical process (diagnostic maneuvers or treatments administered) or outcomes (e.g., fertility, milk yield, complications, cull/survival) were considered, along with level of interest for each topic. During the PSM with all three practices it was evident that participants were keen to find answers to frequently encountered clinical problems, and topic proposals often strayed into areas of practice-based research aimed at answering those clinical questions.

Practice 1 selected two final candidate topics, with a subsequent vote selecting a process audit of cystic ovarian disease (COD) treatment on initial and follow-up visits. Practice 2 team members reached consensus agreement to audit calf pneumonia four-week survival; this topic was later replaced with a process audit of endometritis treatment due to poor case accrual for the first topic. For Practice 3, an outcome (survival, milk yield, and days to conception) audit of left displaced abomasum surgery (LDA) was deemed both feasible and relevant to the practice by the meeting participants.

### 3.3. Clinical Audit Standard Selection

CAB Abstracts and Medline searches identified 128 articles relevant to the treatment of cystic ovarian disease. Multiple treatments of COD were described but no studies or guidelines for current standard of care were identified. For the treatment of endometritis, 99 relevant articles were identified, none of which identified evidence or guidelines for a “gold standard” of care. Seven publications were identified in which LDA surgical outcomes were reported, none of which were clinical practice guidelines. Participating clinicians at Practice 3 agreed to use these seven publications as benchmarks for their clinical audit of LDA surgical outcomes [16,17,18,19,20,21,22].

### 3.4. Potential Sources of Data for Retrospective Clinical Audit

A variety of potential sources of clinical data were available in all three practices but the level of detail necessary to identify cases varied by practice and by data source (Table 2).

### 3.5. Prospective Data Collection

Details of each practice’s prospective clinical audit topic and processes are found in Table 3.

### 3.6. Clinical Audits

#### 3.6.1. Practice 1–Retrospective Clinical Audit

Practice management software (PMS) clinical notes generally included the reason for the visit but additional data varied greatly between veterinary surgeons–not all veterinarians had recorded details about individual animals and clinical findings or treatment plans were often absent. Clinical notes and billing data were examined for all terms and invoice items possibly related to cystic ovary disease but no approach was adequate for case identification. Fertility visit notes were also assessed for case identification: 942 ‘fertility’ visits were recorded, with 20,000 scans but again, no details allowing identification of COD cases were available. Similarly, Interherd was typically used by the practice for herd health problem investigation and COD cases were not routinely recorded. Finally, paper Herd Health Visit Record Sheets (HHVRS) accrued during 2013 were examined. Of legible findings from 198 HHVRS sheets, 89 individual animal records had a written diagnosis of ‘cystic ovarian disease’—in most cases the type of cyst was not specified (75/89) and were presumed to be follicular. However, ~20% of these cases were treated with cloprostenol, suggesting that some of these cases may have been considered luteal cysts (Table 4). In addition to the cases in which cyst type was not specified, 13 cases had a written diagnosis of ‘luteal cyst’ and one was diagnosed as a ‘follicular cyst’. The treating veterinarian was not routinely recorded on the HHVRS data. Data identification and extraction from HHVRS took KW one day to complete.

#### 3.6.2. Practice 1–Prospective Clinical Audit

During prospective clinical audit, 251 examinations recording ovarian findings in 209 individually identified cattle were logged. Three veterinarians accounted for the majority (90.4%) of the examinations and the majority of records were complete. Average number of examinations collected during the first three months of audit (39 examinations/month) declined over the last three months of the audit (20.7 examinations/month). Initial and follow-up examinations were coded by the veterinarians for 202 and 49 examinations, respectively. However, examination of individual cow records indicated that a total of 209 cows were examined with 34 of those seen for one or more follow-up examinations (two visits n = 26, three visits n = 8). Sixteen records coded as follow-up visits were cows seen on initial exam during the clinical audit period while nine rechecks were not appropriately coded. Accurate recording of follow-up examinations was reported to be difficult in practitioner comments during interim communications.

The majority of cows were diagnosed by ultrasound (207/209) with no significant difference in method of diagnosis between the three busiest veterinarians (data not shown). Follicular and luteal cysts were the most common diagnoses recorded with buserelin the most common treatment administered for follicular cysts and cloprostenol most commonly used to treat luteal cysts (Table 4). Dosing was relatively consistent amongst practitioners for buserelin with identical dose administration in 176/185 of doses recorded. However, one veterinarian dosed cloprostenol at 0.5 mg (accounting for 11 of 12 administrations of that dose) whilst other practice veterinarians routinely administered 1.0 mg (18/30 total cloprostenol treatments). Manual rupture was a rare and unintentional occurrence during palpation according to free text comments.

#### 3.6.3. Practice 1–Clinical Audit Satisfaction

Results from both retrospective and prospective clinical audits were communicated with the veterinary surgeons of Practice 1. Just two veterinary surgeons were present during in-person review of the prospectively collected data: the partner who was clinical audit lead, and a new associate who had not been involved from onset. Additional communication with the practice team took place via email. Relative speed and usefulness of data collection from HHVRS was welcomed for the retrospectively collected data, although reliability of information was of concern due to the potential transcription errors during farm visits. Additionally, applicability of data from herd visits two years prior to the time of the prospective collection was questioned due to changing practice staff, client base, and frequency of herd health visits. Participants were satisfied that the prospective clinical audit indicated relatively consistent treatment approaches to COD within the practice. Change in protocols was not viewed as necessary and a decision not to re-run the audit was made by the practice as consequence.

#### 3.6.4. Practice 2–Retrospective Clinical Audit

PMS records from Practice 2 generally contained only chargeable invoice items (time on farm, visit charge, and product sales) with the exception of surgical visits. No clinical details about individual cases were recorded in the PMS. Day book schedules were also insufficiently detailed to allow identification of calf pneumonia cases. Product sales identified that 4715 mL of tulathromycin, the antibiotic most commonly used to treat calf pneumonia in this practice, was dispensed in 2013 but with insufficient detail to allow a clinical audit on use or outcomes. Thus, no data sources with which to perform a retrospective clinical audit were identified.

#### 3.6.5. Practice 2–Prospective Clinical Audits

Clinical audit of calf pneumonia was suspended due to low case accrual (one case in a six month period). As endometritis had been a popular topic during the PSM, a decision was made to replace the original topic with that of endometritis treatment.

Two veterinarians accounted for all of the data collected. Data was collected during four months (July–October), with an additional month of collection in early spring (March). Average number of examinations collected increased over the first three months of the clinical audit (17, 21, 57 cases, respectively) but declined in the fourth month (17 cases). Initial and follow-up examinations were coded by the veterinarians for 85 and 45 examinations, respectively, with one exam not coded. However, inspection of individual cow clinical audit records indicated that a total of 100 cows were examined with 26 of those seen for one or more follow-up examinations (two visits n = 21, three visits n = 5). Seventeen records coded as follow-up visits were cows seen on initial exam during the clinical audit period while three rechecks were not appropriately coded as such. One veterinarian accounted for the majority of exams (118/131) and the majority of records were complete. Median endometritis exams recorded per farm was three (IQR 2-12).

Treatments were administered in 109/131 examinations (Table 5). Cloprostenol (as Cyclix) was used as a sole treatment 77% of the time or in addition to systemic antibiotic (16% of prostaglandin- treated cows) or intrauterine cephapirin (Metricure, 12% of prostaglandin treated cows). Intrauterine cephapirin was the sole treatment used in 24 patients. Increasing purulence of uterine discharge was paralleled by increased use of intrauterine or systemic antibiotic, as did the presence of malodour. The most common systemic antibiotic used was injectable ceftiofur (recorded as Naxcel, 15/17 systemic antibiotic administrations).

#### 3.6.6. Practice 2–Clinical Audit Satisfaction

Results were communicated with the veterinary surgeons of Practice 2 in an in-person meeting which most of the original PSM surgeons attended (80%). With regard to the lack of data for retrospective clinical audit, participants suggested that their shift from itemized charges to time-based billing resulted in probable data loss and that this trend was likely to affect many practices engaged in farm animal work. Additionally, they considered small animal PMS to confer relative advantage for clinical audit due to automated patient ID recording, which would have to be recorded manually for farm animal work in their practice.

Presentation of prospective data stimulated discussion regarding relative efficacy of intrauterine antibiotic suspension and prostaglandin with different endometritis presentations. Final data analysis, as well as literature to answer specific efficacy questions were communicated via an emailed PowerPoint presentation. No further stages of the clinical audit were conducted as decided upon by the practice.

#### 3.6.7. Practice 3–Retrospective Clinical Audit

PMS records were mainly used for billing purposes but clinical notes, though brief, recorded reason for visit as well as charged medications. Although most practice activity was charged by time rather than procedure, LDA surgery was recorded as a chargeable event. Although a list of LDA surgeries, along with time and place of occurrence, could be generated from the PMS, patients were not individually identified thus records could not be linked to follow-up data from Interherd records. Retrospective clinical audit could not be undertaken because of this issue.

#### 3.6.8. Practice 3–Prospective Clinical Audit

During prospective clinical audit, 68 LDA surgical cases were recorded by eight different veterinary surgeons on 27 different farms during a period of eight months (Table 6). Each veterinarian recorded at least 2 cases (median 6 cases, IQR 3-12). Number of cases per month was fairly consistent over the time of the audit (median 6, IQR 3–8, range 3–15). Approximately one third had at least one recorded comorbidity (n = 24) with uterine pathology most common (n = 12). There were no intraoperative deaths. Prognosis was scored for 67/68 cows with 38 scored as ‘good’, 25 as ‘fair’, and 4 as ‘poor’.

Follow-up data was not recorded for all cases and was sparse for milk yield and fertility outcomes. Two of 48 cows for which there was initial survival information died within one week of surgery, with 46 remaining alive. Two-month survival information was available for 41 cows, all of whom remained alive. All four cows given a poor prognostic score at the time of surgery were alive at 2 months; the two patients who died within the first week were given a fair prognosis. Milk yield was not recorded on all farms (13/27 recording): for 25 cows where data was available, mean milk recorded was 35.3 litres (Standard deviation 10.6L) at a mean time of 54 days post-LDA surgery (SD 20 days). Average herd yield from reporting farms was 30.6L (SD 2.87) per cow during this time period. For 26 cows which had fertility information recorded, mean time to first service was 63 days (SD 21 days); for 13 cows in which pregnancy outcomes were recorded, ten conceived (none from first service), one died from an injury and two were culled. Median time from calving to pregnancy diagnosis was 94 days (IQR 83-110).

#### 3.6.9. Practice 3–Clinical Audit Satisfaction

Results were communicated via in-person meetings which half of practice surgeons attended. As LDA surgery continued to be billed as an itemized procedure, many attendees discussed the addition of an individual cow ID field on farm tickets to enable future retrospective clinical audits of existing data.

Participants were satisfied that the outcomes garnered from the prospective clinical audit matched or exceeded benchmarks established in the literature search. A decision by the practice to extend the clinical audit resulted in the collection of 19 additional cases which did not substantially alter the original findings (data not shown). No further stages of clinical audit were conducted.

#### 3.6.10. All Practices–Practitioner Feedback on the Clinical Audit Process

Comments from participants at final clinical audit meetings contained similar themes. In general, participants assessed the experience as interesting and useful, particularly in stimulating discussion of current best evidence for diagnosis and treatment of each condition, as well as reassuring for their own practice. The relationship between clinical audit and research was discussed at length in all final meetings with the suggestion that differentiation is more difficult in situations where there is relatively little clinical evidence. Some expressed an interest in re-running similar audits due to changes in products, processes, and staff members since the initiation of the clinical audits. However, one or more participants expressed concerns regarding time requirements, cost–benefit to the practice, lack of robust evidence against which to compare clinical audit results, decreased staff engagement over longer time periods, biased data by variation in farmer herd health strategies, as well as the possibility of selective outcome recording by clinicians in prospective clinical audits. A number emphasized the importance of a clinical audit leader, as well as short duration clinical audits on simple topics to enhance the likelihood of success and engagement. One practitioner felt that university participation in clinical audit could better align academic veterinary research with veterinarians in private clinical practice and a number of participants were keen to bridge the perceived gap between university and practitioner research priorities.

## 4. Discussion

Clinical audit was adopted from the medical profession in the late 1990s and is now widely discussed as a quality improvement tool in veterinary medicine [1,6]. While there have been prior reports of clinical audit in veterinary medicine, to date this is the most extensive description of the clinical audit process used in farm animal practice. We found that while practitioners were enthusiastic about clinical audit, retrospective clinical audit may be more challenging to undertake in farm animal practice: prospective data collection is feasible but workload, duration, and ease of case accrual and follow-up should be considered when initiating the project.

Clinical audit as performed in human medicine often measures service delivery or outcomes against explicit criteria based on evidence-based standards of care. In this setting ‘research is concerned with discovering the right thing to do whereas audit is intended to make sure that the thing is done right’ [23]. In veterinary medicine, little high-quality evidence exists for many treatments, procedures or outcomes, thus standards or benchmarks are less obvious. Evidence-based standards or guidelines could not be found for any of the clinical audit topics selected by participants. Without defined ‘gold standards’, the ambiguity of clinical audit versus practice-based research was a recurrent topic amongst participants throughout this project. This uncertainty concurs with findings from surveys of farm animal and equine practitioners [24,25]. One source of the confusion may be that clinical audit and clinical research can be very similar and can potentially produce the same, or similar, data and utilize similar data sources and collection methods [26]. The distinction between clinical research and clinical audit are suggested to be driven by sample size, data quality and complexity, ethical review, and external validity [23,26,27] but those contrasts are lessened in veterinary medicine where much of clinical research is comprised of retrospective observational studies from referral populations and where the ‘right thing to do’ is often uncertain [28,29,30].

Prior reports have described the use of existing data for retrospective clinical audit [9,31,32], but no previous publications have examined the implementation of retrospective clinical audits in different farm animal practices. Although there are a number of different ways in which farm animal practices store data, the amount and quality of information found in the three practices was sparse and limited the ability to conduct retrospective clinical audit. Although enough data was found to look at the treatment of COD in Practice 1, the lack of any clinical data in Practice 2 and the lack of patient identification in Practice 3 prohibited any form of retrospective data collection on the chosen topics. The data that was found in Practice 1 was from an unexpected source and results paralleled those from the prospective clinical audit of the same process, though with less diagnostic detail. The ability to conduct retrospective clinical audit will depend on the availability of existing data, and this may be challenging in instances where individual patient identification does not appear to be routinely recorded or linked to clinical notes. Retrospective clinical audit using PMS data may be more feasible in companion animal medicine where unique patient identification is routine and there is institutional support for database management and coding systems; in farm animal practice, alternative sources may be a richer source of data.

Practitioners found prospective data collection forms relatively easy to use and on-farm recording was believed to maximize accuracy. Subsequent transfer to an electronic format was somewhat more time consuming; utilization of a mobile off-line data collection tool could streamline this process [33]. The data collection forms represented a compromise between participants’ desire for detailed information against perceived time constraints and client acceptance of data recording. It is possible that key data collected in all three prospective clinical audits may have been obtained with fewer questions. Initial data quality was high, although complete follow-up data was notably more difficult to obtain, a factor which should be considered in planning outcome audits. Other authors [10] have suggested that data collection for prospective clinical audit is relatively labour intensive and of relatively long duration for adequate case accrual. Our results suggest that workload and duration may be modifiable factors with careful planning of the clinical audit but audit coordination is still a time-intensive activity.

Clinical audit duration was somewhat affected by the logistics of practitioner meetings and speed of case accrual. Practice meetings were sometimes difficult to schedule due to the ambulatory nature of farm animal work and, despite being arranged to maximize attendance, not all participants were able to attend. Case accrual was affected by frequency of presentation and seasonality, and in the case of Practice 1, declined over time. Due to the varying case mix between participants, not all veterinary surgeons were able to equally participate, despite the intention of wide engagement. In Practice 2, there was an explicit trade-off between wider participation and timely accrual which resulted in a change of clinical audit topic. Over the course of this project, a number of staff changes occurred at each of the three practices such that not all initial participants were still with the practice at clinical audit completion and final meetings were attended by new staff who had not previously participated. Lack of staff continuity and adequate authority has been cited as a barrier to clinical audit cycle completion after initial data collection [34].

Time is consistently reported as a barrier to clinical audit [35,36]. Similar to our findings from a national survey of attitudes of farm animal veterinarians towards clinical audit [25], time burden was often raised as an area of concern. Within the NHS, clinical audit is government-supported through both hospital and national audit infrastructure and personnel [34,37,38]. However, even with institutional support, post-graduate medical trainees still view clinical audit as an additional time burden [36]. Veterinary clinical audit must, by necessity, be self-funded by the practice [37] and be performed with little or no governmental support. Veterinary surgeons, particularly those in farm animal practice, are at financial risk and have relatively long duty hours [39,40]. The practitioners in the three practices and in the nationwide survey suggested uncertainty about the cost-effectiveness of clinical audit in farm animal practice [25]. The clinical audits reported here were coordinated by a single researcher (KW) for periods ranging from 11 to 28 months with financial support from the Centre for Evidence-based Veterinary Medicine; no cost accounting of researcher or practice staff time was done but the participants felt that researcher assistance was essential to the performance of their clinical audits.

Relatively large amounts of time and resources have been spent on researching and promoting clinical audit in comparison to other quality improvement methods. Clinical audit has been incorporated into the RCVS Code of Professional Conduct and the RCVS Practice Standards Scheme, it is listed as an RCVS CPD activity and a clinical audit module exists for the RCVS Certificate in Advanced Veterinary Practice. RCVS Knowledge has more recently provided clinical audit training with freely available courses and templates (https://knowledge.rcvs.org.uk/quality-improvement/tools-and-resources/clinical-audit/) and there have been guidance articles produced previously [1,4,5,41,42,43]. Additionally, RCVS Knowledge supports several national small animal audits https://vetaudit.rcvsk.org/ as well as two larger databases of small animal laboratory and clinical records (VetCompass and SAVSNET) [44,45] which may be useful for clinical audit [46,47]. However, to date, institutional support for farm animal clinical audit has primarily been driven by academic centres (as in the case of this work) and private practice (as in the case of a large cattle caesarian section clinical audit performed by a large collaborative group of farm animal veterinary practices [48,49]). Use of electronic databases has been proposed as a path forward in supporting both clinical audit and of complementary clinical research but similar infrastructure and support is not currently available for farm animal practice. In this study, we found that lack of individual patient identification in PMS can hinder retrospective clinical audit in farm animal practice, although other data sources may be available. In some countries with nationalized herd management systems and coding systems, data for retrospective clinical audit may be more easily collected by veterinary surgeons [50]. However, for many farm animal clinicians, digital and identifiable outcome data may be siloed in private herd management databases which may not be as accessible unless the surgeon is a contractor or employee. In contrast, prospective clinical audit proved feasible and our findings highlighted that consideration of case accrual, user-friendly data collection tools, and ability to collect longitudinal data should be incorporated in prospective clinical audit planning.

### Limitations

It cannot be assumed that the findings of this study represent the opinions and experiences of all veterinary surgeons that undertake farm animal work in the UK. All three practices had volunteered to work on this project and therefore these veterinary surgeons may have been more motivated than their peers to undertake clinical audit. Similar audits carried out in practice settings resourced with mobile veterinary software and/or widely implemented automated herd health recording might use different processes with improved efficiencies. Similarly, clinical audit performance when coordinated by an outside researcher might vary from that coordinated by internal personnel if any presumed Hawthorne effect [51] is mediated by perceived observer status; moreover, if a Hawthorne effect is present during any clinical audit, results may not fully parallel what happens in unobserved practice.

## 5. Conclusions

Although a number of barriers to conducting clinical audit in farm animal veterinary practice were highlighted by this study, it was demonstrated that clinical audit in this environment is feasible. Participating veterinary surgeons were happy and willing to engage in clinical audit activities; although retrospective clinical audit was challenging, it was possible for data to be collected prospectively. Clinical audit topics which require linkage of patient data over more than one encounter may be more difficult to implement in farm animal practice than in companion animal practice because of differences in patient data recording. Despite these barriers, this study found that conducting clinical audit can be of benefit to practitioners, particularly the opportunity to discuss best available evidence, a topic of keen interest to participants. However, institutional support for clinical audit training adapted to farm animal practice, as well as data collection from laboratory networks and herd health information systems capturing production animal health data may be important.

## Figures and Tables

**Table 1:** Topics considered and selected for clinical audit in each practice.

Topic	Practice 1	Practice 2	Practice 3
Calf pneumonia		Outcome audit (4 week survival) Discontinued due to low case accrual	
Calf scours (pre-weaning)			
Cystic ovaries	Process audit (treatments administered)		
Bovine eye diseases			
Bovine acidosis			
Digit amputation			
Dystocia			
Endometritis		Process audit (treatments administered)	
Ketosis			
Left displaced abomasum correction			Outcome audit (survival, fertility, milk yield)
Metritis			
Tilmicosin for drying off			
Retained fetal membranes			
Right-sided ping			
Toxic mastitis			

Light grey shading indicates initial topics nominated by the practice; boxed topics were those selected to take forward as clinical audit topics.

**Table 2:** Sources of information identified for possible retrospective data collection within the 3 practices undertaking a farm animal related clinical audit.

Practice	Practice Management Software	Herd Records	Daily Schedule	Invoice Items
Practice 1	Robovet	Interherd software Herd Health Visit Record Sheets	Online day book	Transferred from paper ticket to PMS
Practice 2	RxWorks	Herd Health Visit Record Sheets	Paper day book	Transferred from paper ticket to PMS
Practice 3	Robovet	Interherd software	Online day book	Transferred from paper ticket to PMS

PMS = practice management software.

**Table 3:** Details of the three practices relevant to the prospective clinical audits carried out by each one over the course of the research.

	Practice 1	Practice 2 First Audit	Practice 2 Second Audit	Practice 3
Topic to audit	COD treatment	Calf pneumonia survival	Endometritis treatment	LDA outcomes
Audit type	Process	Outcome	Process	Outcome
Initial data collection	Paper form	Paper form	Paper form	Paper form
Follow-up data collection	Not applicable	Farmer communication	Not applicable	Interherd
Participating veterinarians	8	6	6	9
Recording veterinarians	6	1	2	8
Participating farms	27	0	13	27
Months of data collection	8.5	6	4.5	8.5
Examinations recorded	251	1	131	68
Total months of audit (Topic selection to end of data collection for analysis)	13	N/A (discontinued)	10.5	12.5

COD = cystic ovarian disease; PMS = practice management software.

**Table 4:** Recorded diagnoses and treatments during Practice 1 clinical audits.

	Retrospective	Prospective			
Diagnosis	‘Cystic’	Follicular Cyst	Luteal Cyst	Corpus Luteum	Uncertain Cyst
Total findings	75	206	31	12	3
Treatments recorded					
Buserelin ^1^	47	178	4	1	2
Cloprostenol ^2^	16	3	23	7	0
PRID ^3^	3	14	0	0	0
CIDR ^4^	4	8	3	1	1
HCG ^5^	0	1	0	0	0
Manual rupture	2	6	0	0	0

^1.^ Recorded as Receptal (MSD Animal health) in retrospective audit, Veterelin (Laboratorios Calier, SA) in prospective audit; 5 mL noted in 86% of retrospectively recorded and 97% of prospectively recorded doses.^2.^ Recorded as Estrumate (MSD Animal health) in retrospective audit for all but one patient in which Cyclix (Virbac Limited) was recorded; recorded solely as Cyclix in prospective audit; 2 mL noted in 21% of retrospectively recorded and 40% of prospectively recorded doses; 4 mL noted in 79% of retrospectively recorded and 60% of prospectively recorded doses. ^3.^ Progesterone Releasing Intravaginal Device (PRID Delta, 1.55 g progesterone, Ceva Animal Health) ^4.^ Controlled Internal Drug Release insert (1.38 g progesterone, Zoetis UK Limited) ^5^ Human chorionic gonadotropin (Chorulon, MSD Animal Health UK); recorded as 10 mL.

**Table 5:** Recorded findings and treatments administered during the endometritis prospective clinical audit by veterinarians in Practice 2 over a 4.5-month period.

			Treatments Administered			
		Total Findings	Cloprostenol ^1^	Buserelin ^2^	Cephapirin Uterine Suspension ^3^	Systemic Antibiotic
**Discharge characteristics**	Clear mucus	21	1	0	0	0
	Clear mucus with flecks of pus	25	16	3	4	1
	Discharge with < 50% pus	37	25	2	11	6
	Discharge with > 50% pus	48	33	1	18	10
**Discharge odor**	No odor	77	49	5	23	9
	Malodorous	23	14	0	9	5
**Corpus luteum**	Present	74	72	0	9	12
	Absent	57	3	6	24	5

^1.^ Recorded as Cyclix (Virbac Limited) 2 mL. ^2.^ Recorded as Veterelin (Laboratorios Calier, SA) 5 mL. ^3.^ Recorded as Metricure (MSD Animal Health).

**Table 6:** Recorded findings from the LDA surgery clinical audit carried out by Practice 3 veterinarians over an 8-month period.

		Veterinarian Code							
		1	2	3	4	5	6	7	8
**Total cases recorded**		3	3	11	24	8	13	4	2
**Rating assigned:**	*Good prognosis*	1	3	6	14	2	9	3	0
	*Fair prognosis*	2	0	4	9	5	3	1	1
	*Poor prognosis*	0	0	1	1	1	0	0	1
	*Prognosis not reported*	0	0	0	0	0	1	0	0
**Outcome 1 week:**	*Survival 1 week*	3	2	5	15	6	9	4	2
	*Death 1 week*	0	0	0	1	0	1	0	0
	*No survival data 1 week*	0	1	6	8	2	3	0	0
**Outcome 2 months:**	*Survival 2 months*	2	2	5	13	6	8	3	2
	*No survival data 2 months*	1	1	6	10	2	4	1	0
**Cases with milk recording data available**		1	2	3	6	2	6	1	1
**Mean milk yield (L)**		27.1	27.3	36.5	34.4	49.2	40.75	31.2	5.8
**Cases with service data**		1	2	2	7	3	9	2	0
**Cases with conception data**		0	1	0	5	1	2	1	0

## Data Availability

Restrictions apply to the availability of these data. Data was obtained from the three participating practices and are available from the authors with the permission of those practices.
